# The impact of marital status at diagnosis on cancer survival in patients with differentiated thyroid cancer

**DOI:** 10.1002/cam4.778

**Published:** 2016-06-05

**Authors:** Rong‐liang Shi, Ning Qu, Zhong‐wu Lu, Tian Liao, Yi Gao, Qing‐hai Ji

**Affiliations:** ^1^ Department of Head and Neck SurgeryFudan University Shanghai Cancer CenterShanghai200032China; ^2^ Department of OncologyShanghai Medical CollegeFudan UniversityShanghai200032China; ^3^ Department of General SurgeryMinhang HospitalFudan UniversityShanghai201199China; ^4^ Department of UltrasonographyFudan University Shanghai Cancer CenterShanghai200032China

**Keywords:** Differentiated thyroid cancer, marital status, SEER, survival analysis

## Abstract

Previous studies have revealed that marital status influences the prognosis of patients with various types of cancer. We evaluated the influence of marriage on the survival outcomes in differentiated thyroid cancer (DTC). The Surveillance, Epidemiology and End Results (SEER) database between 2002 and 2012 was used to compare cancer‐specific mortality in different marital status, and in each sex, age, and stage stratification by multivariate Cox regression model. In total, 61,077 eligible patients were identified. The widowed group had the highest proportion of women, elderly patients (≥45 years), and advanced stage III/IV tumor (*P *=* *0.001), but the total thyroidectomy (TT) performed and radioisotopes therapy rates were lower than those in the married group. Married patients had a better cancer‐specific survival (CSS) than the unmarried (*P *<* *0.05). Further analysis showed that widowed patients always presented the lowest CSS compared with other groups. Widowed patients had a significant increased risk for CSS compared with married patients in males [hazard ratio (HR) 2.72, 95% confidence interval (CI): 1.59–4.65, *P *=* *0.001], females (HR 2.02, 95% CI: 2.24–4.06, *P *=* *0.001), young patients (<45, HR 28.12, 95% CI: 3.48–227.25, *P *=* *0.002), elderly patients (≥45, HR 28.12, 95% CI: 2.97, 95% CI: 2.30–3.83, *P *=* *0.001), stage I (HR 8.44, 95% CI: 4.05–17.59, *P *=* *0.001), stage II (HR 3.64, 95% CI: 1.30–10.20, *P *=* *0.014), stage III (HR 2.27, 95% CI: 1.08–4.78, *P *=* *0.031), and stage IV (HR 2.63, 95% CI: 1.94–3.57, *P *=* *0.001). These results showed that unmarried status, especially for widowhood, increased the risk of cancer mortality in DTC patients.

## Introduction

Thyroid cancer is the most common endocrine malignancy. Approximately 62,980 cases of thyroid cancer were newly diagnosed in the United States in 2014, and the number continues to increase annually 1. Much attention is focused on differentiated thyroid cancer (DTC), a disease that accounts for more than 90% of thyroid cancers but tends to have an indolent clinical course with low morbidity and mortality 2, 3. Survival analysis in the study by Davies and Welch indicates that mortality rates for thyroid cancer have remained steady at 0.5 deaths per 100,000^4^, ^5^. More specific studies of the SEER database utilizing relative survival show different thyroid cancer survival rates based on sex, ethnicity, tumor histology, and stage 6. The 5‐year relative survival rates have increased among women, from 92.7% in 1974 to 97.4% in 2001^6^. However, the annual percentage change (APC) in thyroid cancer mortality rates in men increased by 2.4%, which is among the largest increases in cancer mortality rates of men in the United States 7. Mortality rates are also affected by race, tumor features, and treatments. 8, 9, 10, 11. Generally, the patients with DTC present a favorable prognosis except for the delayed diagnosis and advanced stage at the time of initial therapy.

It is well known that social support and environmental factors may influence overall mental state of well‐being, so as to exerting a significant effect on the outcome, especially for cancer patients. It has been shown that married persons have better mood and receive more social support, including practical support and financial resources, so that they can focus on treatment and generally show a better recovery from a single malignancy. In all of the top 10 causes of cancer death in the United States, the survival benefit associated with marriage was even larger than the published survival benefit from chemotherapy in prostate, breast, colorectal, and esophageal cancers 12, 13. However, little is known with respect to the prognostic significance of marital status in DTC patients. A better understanding of the impact of marital status on thyroid cancer patients would give more support on the importance of social mechanisms in management of this endocrine malignancy and help establish a more holistic approach that may improve patient outcomes. In this study, we extracted data from the Surveillance, Epidemiology and End Results (SEER) cancer‐registry program of individuals diagnosed as DTC between 2002 and 2012 to explore the relationship between a certain marital status and the survival of patients in detail.

## Methods

### Patient selection from the SEER database

We extracted data from the SEER cancer registry to conduct this study. SEER, a population‐based registry sponsored by the National Cancer Institute, collects information on cancer incidence and survival from 17 population‐based cancer registries, including approximately 28% of the US population 14. SEER data contains no identifiers and is publicly available for studies on cancer‐based epidemiology and health policy. The National Cancer Institute's SEER*Stat software (Surveillance Research Program, National Cancer Institute SEER*Stat software, www.seer.cancer.gov/seerstat) (Version 8.1.2) was used to identify patients who were diagnosed from 2002 to 2012, with single primary DTC. Patients with surgical and postoperative radioactive therapy for PTC were included. Histology types were limited to papillary carcinoma and its variants (8250, 8260, 8330, 8331, 8332, 8335, 8340, 8341, 8343, 8344). The patients were excluded if with: insufficient or unknown clinicopathologic‐profile, short follow‐up period (<6 months), primary tumor rather than the above mentioned histologies, undetermined histology or other types of thyroid cancer (medullary and anaplastic carcinoma). The patients with papillary squamous cell carcinoma and oxyphilic variant were excluded due to the rarity that affected the efficiency of statistic analysis.

### Clinicopathological variable assessment

The variable of “marital status” referred to “the status at diagnosis” when not otherwise specified. Marital status was coded as married, divorced, widowed, and single (never married). Age, sex, race, histological subtype, TNM stage, survival time, and cause‐specific survival (CSS) were extracted from the SEER database. Race was categorized into African American, non‐Hispanic white, and others (American Indian/AK Native, Asian/Pacific Islander) as provided by the SEER database.

We followed the guidance of the 2010 TNM classification of American Joint Committee on Cancer/International Union Against Cancer 15, 16, 17. The endpoint of present study was CSS which was calculated from the date of diagnosis to the date of cancer‐specific death and was shown as “SEER cause‐specific survival” in SEER database.

### Statistical analysis

Chi‐squared (*χ* ^2^) test was used to compare patients' baseline characteristics. Survival rate was generated using Kaplan–Meier curve, and the differences were compared with the log‐rank test. Multivariate Cox regression models were applied to determine whether there was an association between marital status and CSS in patients receipt of definitive treatment, adjusted for patient demographics (as listed above) and tumor characteristics (tumor histology classification)^18^.

Cox proportional hazards regression model was then built to evaluate the impact of marital status on cancer mortality from DTC among patients according to sex, age, and TNM stage after being adjusted for the previously listed variables as well as receipt of definitive therapy. Finally, the cumulative incidence of cancer‐specific mortality stratified by marital status was generated from Kaplan–Meier curve models described above and displayed graphically.

The hazard ratio (HR) for relationships between each variable and mortality was calculated using binary Cox regression model. All confidence intervals (CIs) were stated at the 95% confidence level. All *P* values were two‐sided. *P *<* *0.05 was considered statistically significant. Statistical analysis was performed using SPSS software, version 13.0 (SPSS Inc., Chicago, IL).

## Results

### Patient characteristics

A total of 61,077 eligible patients were identified during the 10‐year study period, including 13,388 male and 47,689 female patients. Among these patients, 40,527 (66.4%) were married, 2,768 (4.5%) were widowed, and 12,889 (21.1%) had never married. The 4,893 (8.0%) individuals who were divorced or separated were grouped together in the divorced group in our study. The widowed group had the highest proportion of women, most prevalent of elderly patients (≥45 years), most common of follicular subtype, most advanced stage III/IV tumor, and most distant metastasis, all of which were statistically significant (*P *<* *0.001). In addition, the widowed group was older than any other marital status subgroup. The rates of total thyroidectomy (TT) and radioisotope therapy performed slightly varied between the married and widowed groups, but both were higher than those in the never married and divorced groups. Patient demographics and clinicopathological features are summarized in Table 1.

**Table 1:** Clinicopathologic characteristics of DTC patients according to marital status in SEER database

Characteristic	All patients no. (%)	Marital status				
		Married no. (%)	Widowed no. (%)	Never married no. (%)	Divorced no. (%)	*P*‐value
No. of patients	61077 (100.0)	40527 (66.4)	2768 (4.5)	12889 (21.1)	4893 (8.0)	
Sex						
Male	13388 (21.9)	9798 (24.1)	256 (9.2)	2547 (19.8)	787 (16.1)	0.001
Female	47689 (78.1)	30729 (75.8)	2512 (90.8)	10342 (80.2)	4106 (83.9)	
Age (mean ± SD)	47.7 ± 14.8	48.9 ± 12.9	68.3 ± 11.9	38.0 ± 15.2	51.5 ± 12.1	
<45	25975 (42.5)	15937 (39.3)	108 (3.9)	8523 (66.1)	1407 (28.8)	0.001^b^
Mean ± SD	33.8 ± 7.6	36.1 ± 5.7	38.0 ± 5.4	29.0 ± 8.6	37.0 ± 5.9	
≥45	35102 (57.5)	24590 (60.7)	2660 (96.1)	4366 (33.9)	3486 (71.2)	
Mean ± SD	57.9 ± 9.6	57.1 ± 9.0	69.6 ± 10.4	55.5 ± 8.5	57.4 ± 8.6	
Race						
Black	3869 (6.3)	1763 (4.4)	238 (8.6)	1383 (10.7)	485 (9.9)	0.001
White	56566 (92.6)	38351 (94.6)	2515 (90.9)	11329 (87.9)	4371 (89.3)	
Other^a^	642 (1.0)	413 (1.0)	15 (0.5)	177 (1.4)	37 (0.8)	
Histology type						
Papillary	57427 (94.0)	38273 (94.4)	2535 (91.6)	12042 (93.4)	4577 (93.5)	0.001
Follicular	3650 (6.0)	2254 (5.6)	233 (8.4)	847 (6.6)	316 (6.5)	
T stage						
T1/T2	47104 (77.1)	31658 (78.1)	1969 (71.1)	9649 (74.9)	3828 (78.2)	0.001
T3/T4	13973 (22.9)	8869 (21.9)	799 (28.9)	3240 (25.1)	1065 (21.8)	
N stage						
N0	46913 (76.8)	31637 (78.1)	2274 (82.2)	9021 (70.0)	3981 (81.4)	0.001
N1a	7098 (11.6)	4536 (11.2)	223 (8.1)	1905 (14.8)	434 (8.9)	
N1b	7066 (11.6)	4354 (10.7)	271 (9.8)	1963 (15.2)	478 (9.8)	
Distant Metastasis	1602 (2.6)	937 (2.3)	122 (4.4)	428 (3.3)	115 (2.4)	0.001
TNM Stage						
I	44537 (72.9)	29209 (72.1)	1469 (53.1)	10540 (81.8)	3319 (67.8)	0.001
II	5088 (8.3)	3380 (8.3)	337 (12.2)	880 (6.8)	491 (10.0)	
III	6991 (11.4)	4871 (12.0)	543 (19.6)	915 (7.1)	662 (13.5)	
IV	4461 (7.3)	3.67 (7.6)	419 (15.1)	554 (4.3)	421 (8.6)	
Surgery procedures						
Lobe.±Isth.	8769 (14.4)	5840 (14.4)	504 (18.2)	1698 (13.2)	727 (14.9)	>0.05
Lobe.±Partial Contra‐lobe.	457 (0.7)	305 (0.8)	29 (1.9)	79 (0.6)	44 (0.9)	
TT or near TT	51851 (84.9)	34382 (84.8)	2235 (80.7)	11112 (86.2)	4122 (82.4)	
Adjuvant therapy						
None	28158 (46.1)	18651 (46.0)	1468 (53.0)	5717 (44.4)	2322 (47.5)	>0.05
Radioisotopes	30512 (50.0)	20322 (50.1)	1167 (42.2)	6656 (51.6)	2367 (48.4)	
Beam radiation	2407 (3.9)	1554 (3.9)	133 (2.8)	516 (2.0)	204 (2.1)	

DTC, differentiated thyroid cancer; lobe., lobectomy; isth., isthmectomy; TT, total thyroidectomy; SD, standard deviation. Data are presented as *n* (%).

a Including American Indian/AK Native, Asian/Pacific Islander.

b *P*‐value refers to comparison for the differences in proportions among subgroups.

### Cancer‐specific survival post initial treatment by marital status on CSS in the SEER database

**Figure 1:**
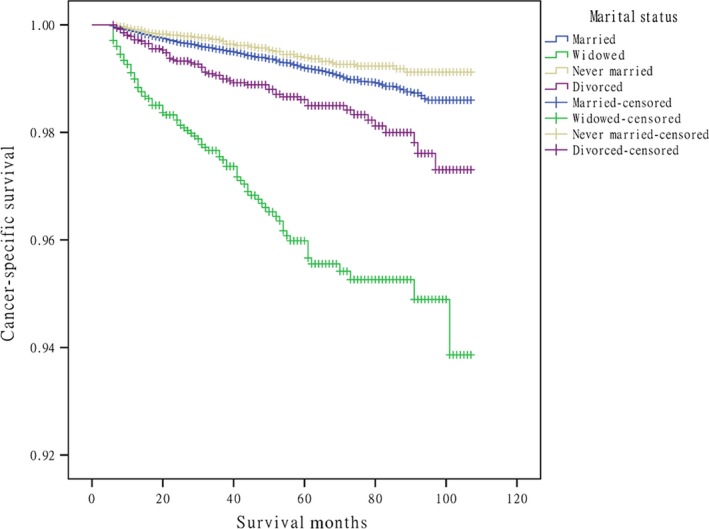
Survival curves in differentiated thyroid cancer patients according to marital status. (*χ*2 = 251.470, *P *=* *0.001).

Married patients with DTC had a better CSS than those unmarried (*P *=* *0.001). Specifically, the overall 10‐year CSS was 97.3% in the married group, 93.9% in the widowed group, 99.6% in the never married group, and 97.3% in the divorced group, which were all significantly different according to the univariate log‐rank test (*P *=* *0.001) (Fig. 1). For clinicopathological variables, sex, age, histological subtype, T/N/M stage, adjuvant therapy, and marital status were identified as independent factors for predicting CSS on univariate analysis (Table 2). When multivariate analysis with Cox regression was performed, the variables which were validated as independent prognostic factors included: sex (female, HR 0.67, 95% CI: 0.55–0.82), age (≥45 years, HR 13.53, 95% CI: 8.65–21.18), tumor stage (T3/4, HR 5.00, 95% CI: 3.97–6.28), node stage (N1a, HR 1.71, 95% CI: 1.29–2.28; N1b, HR 1.77, 95% CI: 1.39–2.26), metastasis (distant, HR 6.65, 95% CI: 5.29–8.35), adjuvant therapy (beam radiation, HR 5.50, 95% CI: 4.15–7.30), and marital status (widowed, HR 2.95, 95% CI: 2.28–3.81; divorced, HR 1.78, 95% CI: 1.34–2.37).

**Table 2:** Univariate and multivariate survival analysis for the influence of marital status on CSS in DTC from SEER database

Variable	Univariate analysis		Multivariate analysis	
	HR (95% CI)	*P*	HR (95% CI)	*P*
Sex (Female vs. Male)	0.37 (0.31–0.44)	0.001	0.67 (0.55–0.82)	0.001
Age (≥45 vs. <45)	16.83 (10.87–26.07)	0.001	13.53 (8.65–21.18)	0.001
Race (White vs. Black)	1.39 (0.90–2.12)	0.135	1.32 (0.86–2.05)	0.206
Follicular vs. Papillary	2.21 (1.69–2.88)	0.001	1.30 (0.97–1.74)	0.073
T3/4 vs. T1/2	10.34 (8.40–12.72)	0.001	5.00 (3.97–6.28)	0.001
N stage				
N0	Reference		Reference	
N1a	2.41 (1.84–3.15)	0.001	1.71 (1.29–2.28)	0.001
N1b	5.82 (4.79–4.07)	0.001	1.77 (1.39–2.26)	0.001
Distant Metastasis	23.65 (19.64–28.49)	0.001	6.65 (5.29–8.35)	0.001
Surgery Procedures				
Lobe.±Isth.	Reference		Reference	
Lobe.±Partial Contra‐lobe.	0.61 (0.15–2.48)	0.484	0.48 (0.12–1.96)	0.305
TT or near TT	1.27 (0.96–1.67)	0.092	0.76 (0.57–1.01)	0.056
Adjuvant therapy				
None	Reference		Reference	
Radioisotopes	1.94 (1.55–2.42)	0.001	1.19 (0.94–1.51)	0.15
Beam radiation	21.72 (16.73–28.20)	0.001	5.50 (4.15–7.30)	0.001
Marital Status				
Married	Reference		Reference	
Widowed	4.98 (3.91–6.34)	0.001	2.95 (2.28–3.81)	0.001
Never married	0.69 (0.52–0.92)	0.011	1.10 (0.83–1.47)	0.500
Divorced	1.83 (1.38–2.43)	0.001	1.78 (1.34–2.37)	0.001

DTC, differentiated thyroid cancer; CSS, cancer‐specific death; lobe., lobectomy; isth., isthmectomy; TT, total thyroidectomy.

### Subgroup analysis for evaluating the effect of marital status according to sex, age, and TNM stage

**Figure 2:**
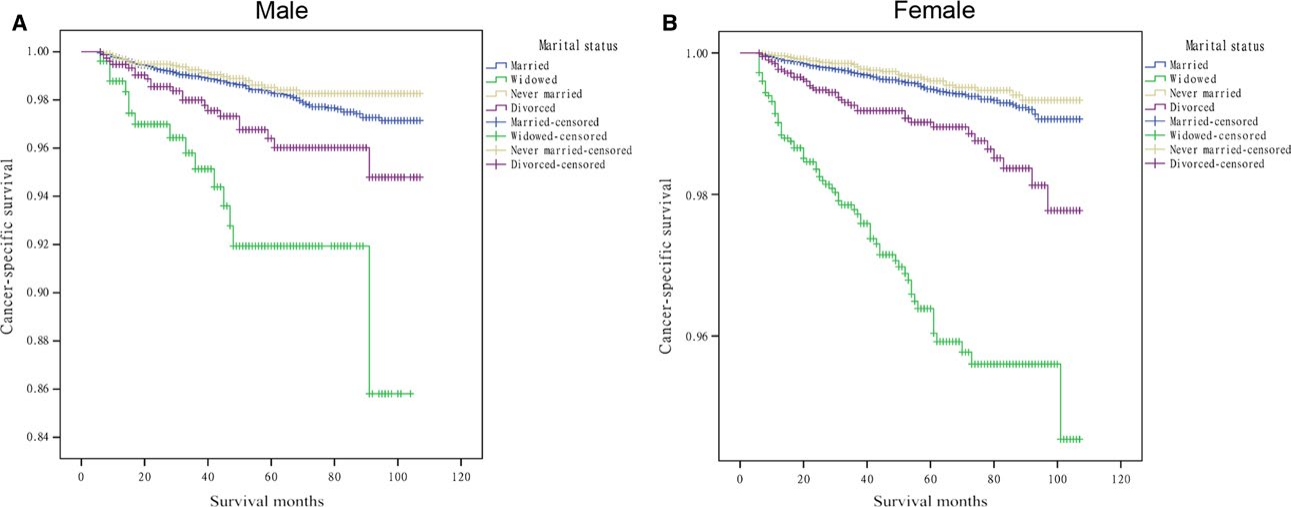
Survival curves in differentiated thyroid cancer patients according to marital status in males and females. (A) Male: *χ*2 = 49.840, *P *=* *0.001; (B) Female: *χ*2 = 282.741, *P *=* *0.001.

**Figure 3:**
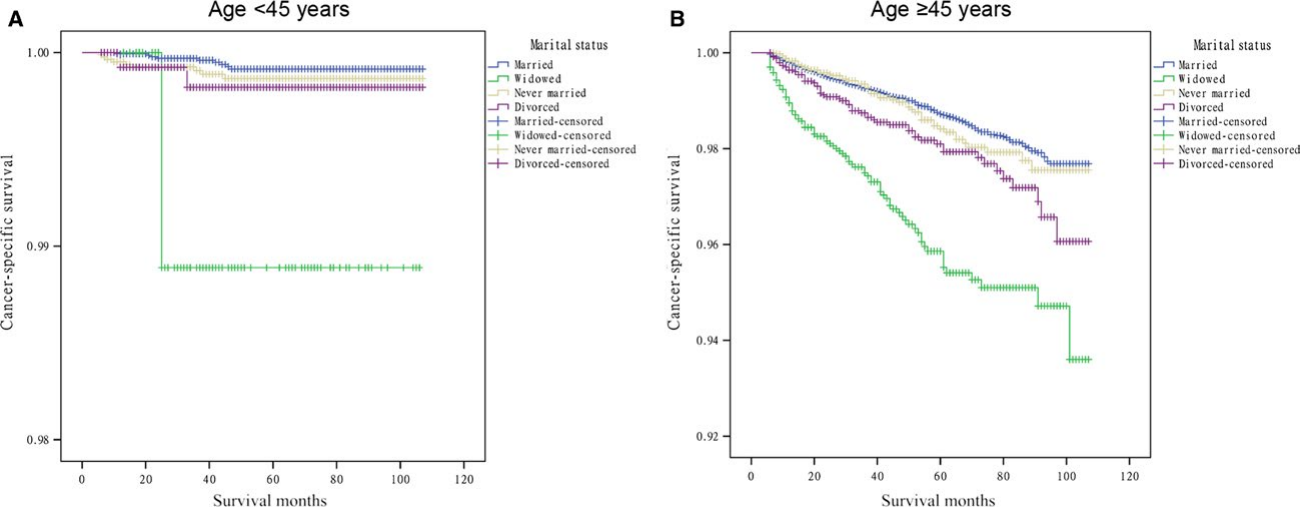
Survival curves in differentiated thyroid cancer patients according to marital status in youths and the elderly. (A) <45 years: *χ*2 = 11.717, *P *=* *0.008; (B) ≥45 years: *χ*2 = 98.826, *P *=* *0.001.

**Figure 4:**
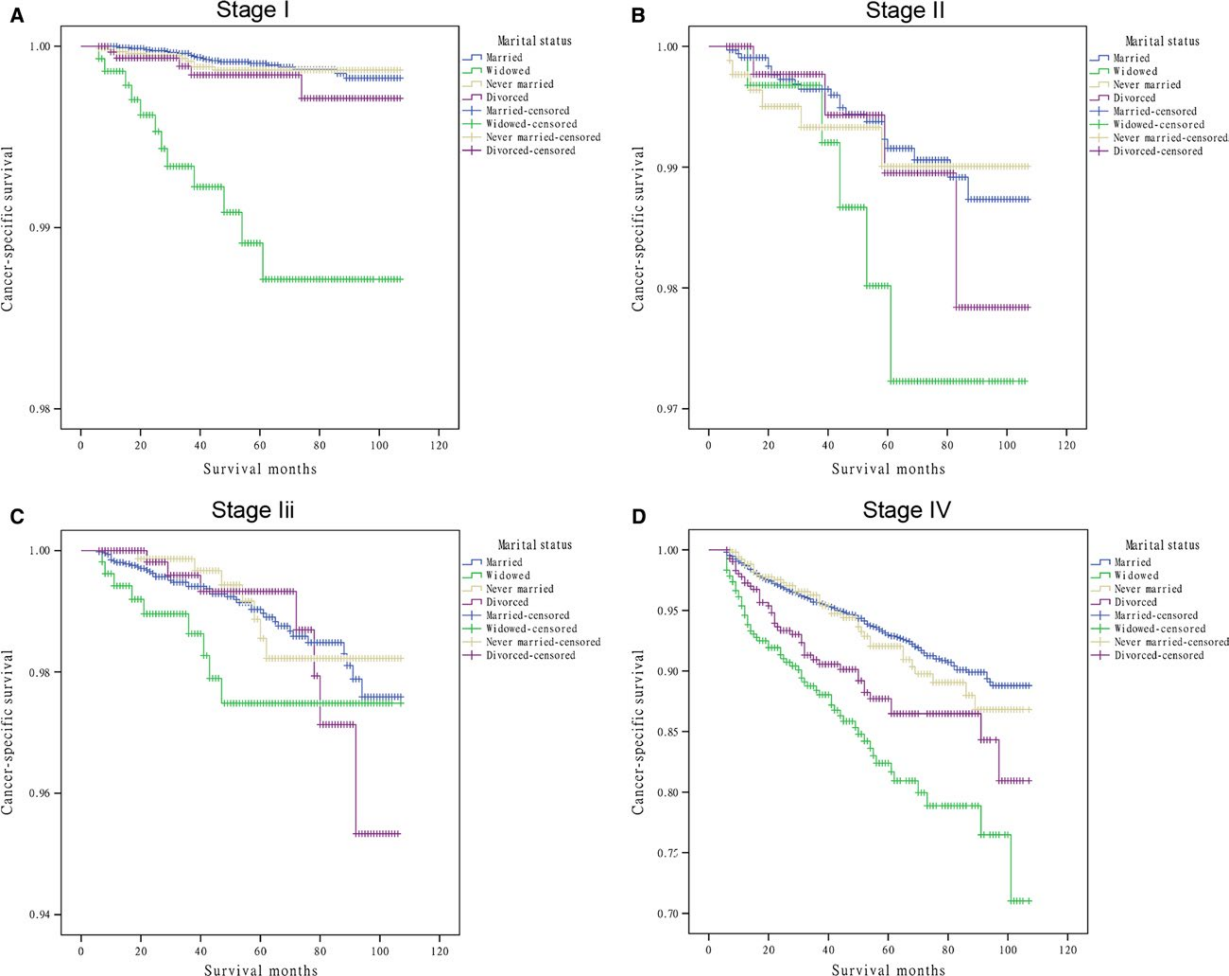
Survival curves in differentiated thyroid cancer patients according to marital status in different stages. (A) Stage I: *χ*2 = 71.886, *P *=* *0.001; (B) Stage II:* χ*2 = 3.496 *P *=* *0.321; (C) Stage III:* χ*2 = 4.186, *P *=* *0.242; (D) Stage IV:* χ*2 = 55.244, *P *=* *0.001.

Results from the Cox proportional hazards regression models identified and verified a series of risk predictors for mortality in DTC, including sex, age, and advanced stage, which were consistent with previous studies 19, 20. Then, we conducted subgroup analysis for the effects of marital status on survival in patients stratified by them. Using the proportional hazards regression models, we found that: 1) being widowed was associated with poorer survival than being married at each sex and age subgroup and all stages of cancer after being adjusted for the aforementioned variables and definitive treatments (*P *=* *0.001); 2) being divorced increased the risk for mortality compared with being married in males (HR 1.81, 95% CI: 1.14–2.87), female (HR 1.73, 95% CI: 1.21–2.49), elderly patients (≥45, HR 1.74, 95% CI: 1.30–2.32) and stage IV cancers (HR 1.90, 95% CI: 1.35–2.65); 3) the difference between the never married and married group was not apparent at each subgroup. (Table 3, Fig. 2, 3, 4).

**Table 3:** Univariate and multivariate analysis of marital status on CSS in DTC according to sex, age and cancer stage

Variable	Univariate analysis		Multivariate analysis	
	HR (95% CI)	*P*	HR (95% CI)	*P*
Sex				
Male				
Married	Reference		Reference	
Widowed	4.61 (2.71–7.85)	0.001	2.72 (1.59–4.65)	0.001
Never married	0.75 (0.50–1.13)	0.173	1.24 (0.81–1.88)	0.327
Divorced	1.94 (1.22–3.06)	0.005	1.81 (1.14–2.87)	0.011
Female				
Married	Reference		Reference	
Widowed	7.15 (5.36–9.54)	0.001	2.02 (2.24–4.06)	0.001
Never married	0.74 (0.50–1.09)	0.126	1.13 (0.76–1.67)	0.553
Divorced	2.22 (1.54–3.18)	0.001	1.73 (1.21–2.49)	0.003
Age				
<45				
Married	Reference		Reference	
Windowed	15.96 (2.02–126.00)	0.001	28.12 (3.48–227.25)	0.002
Never married	1.90 (0.75–4.78)	0.174	1.46 (0.57–3.74)	0.436
Divorced	2.50 (0.54–11.57)	0.241	2.92 (0.62–13.72)	0.176
≥45				
Married	Reference		Reference	
Widowed	3.18 (2.49–4.05)	0.001	2.97 (2.30–3.83)	0.001
Never married	1.11 (0.82–1.50)	0.503	1.11 (0.82–1.50)	0.518
Divorced	1.57 (1.18–2.09)	0.002	1.74 (1.30–2.32)	0.001
TNM stage				
Stage I				
Married	Reference		Reference	
Widowed	10.82 (5.38–21.74)	0.001	8.44 (4.05–17.59)	0.001
Never married	1.22 (0.5802.56)	0.602	1.74 (0.81–3.78)	0.159
Divorced	1.95 (0.74–5.11)	0.178	1.87 (0.71–4.95)	0.208
Stage II				
Married	Reference		Reference	
Widowed	2.46 (0.93–6.52)	0.071	3.64 (1.30–10.20)	0.014
Never married	1.21 (0.49–3.00)	0.670	1.08 (0.41–2.84)	0.879
Divorced	1.32 (0.45–3.85)	0.610	1.64 (0.56–4.82)	0.366
Stage III				
Married	Reference		Reference	
Widowed	2.01 (0.98–4.12)	0.058	2.27 (1.08–4.78)	0.031
Never married	0.88 (0.39–1.95)	0.749	0.92 (0.41–2.05)	0.829
Divorced	1.20 (0.54–2.66)	0.665	1.23 (0.55–2.76)	0.613
Stage IV				
Married	Reference		Reference	
Widowed	2.73 (2.05–3.65)	0.001	2.63 (1.94–3.57)	0.001
Never married	1.13 (0.79–1.62)	0.501	1.07 (0.75–1.54)	0.710
Divorced	1.802 (1.29–2.51)	0.001	1.90 (1.35–2.65)	0.001

DTC, differentiated thyroid cancer; CSS, cancer‐specific death. *P* values refer to comparisons between two groups and were adjusted for age, race, histologic type, surgery procedures, as covariates. NI: not included in the multivariate survival analysis.

## Discussion

Dating back to the 19th century, the literatures showed that married persons had decreased death rates from a variety of diseases 21. Cancer is a major public health problem on a global scale. One in four deaths in the United States is due to cancer 7. In the United States, it is also reported that 51% of Americans are married 13. Therefore, the presence of a consistent association between marital status and outcome across multiple cancers would support the notion that unmarried patients with any malignancy represent a high‐risk population that might benefit from targeted, support‐based interventions 13. However, current studies assessing the impact of marital status on CSS among patients with cancer have presented conflicting results, and few of them have investigated the effects of marital status on patient survival or focused on the heterogeneity of unmarried patients in DTC. This study was designed to determine how marital status at diagnosis affected cancer mortality in the entire cohort and all categories were stratified by traditional prognostic factors, such as sex, age, and AJCC cancer stage.

In the analyses conducted thus far, it was indicated that marital status was an independent prognostic factor in DTC patients; additionally, the increased relative risks of DTC death was significant among widowed patients compared with married patients at each sex, age subgroup and all stages of cancer. The results were similar with previous researches investigating the associations between marital status and survival in breast, lung, colorectal, esophageal, and gastric cancers. 22, 23, 24, 25. While the mechanism underlying the improved survival associated with marriage in patients is not entirely clear, with the analyses from this study, the possible reasons for the unfavorable prognosis of unmarried patients, especially for widowed or divorced ones, are proposed as follows. First, widowed or divorced patients tend to be diagnosed as DTC at an elderly age. Age is considered to be the most important prognostic factor as demonstrated by the majority of current risk stratification systems. To the best of our knowledge, TNM classification is the only staging system that adopts 45‐year as the cut‐off value for age to stratify the patients in high‐ and low‐risk groups for cancer‐specific mortality 16, 17. The fact that the widowed group has the highest proportion of elderly patients (≥45 years) and greatest overall mean age becomes one hypothesis to explain the influence of marital status on prognosis in DTC. Second, widowed or divorced patients, tend to be diagnosed at a late stage of disease. The high incidence of advanced stage III/IV tumor and distant metastasis in the widowed group suggests a spread of primary tumor that conferred to a poor prognosis; however, the rates of surgical resection (TT performed) and radioisotopes therapy are both the lowest in the widowed group, which is a reflection for undertreatment of definitive or potentially curative therapies in these patients.

It is noteworthy that our results were consistent with previous studies in which married patients with head and neck cancer had the greatest relative reduction in cancer‐related death compared with unmarried patients 12, 13. Interestingly, recent data also reported that the impact of marital status on mortality varied with sex and age in some cancers 23, 26. As sex and age are known to possibly interact with marital status in a general population, we further tested associations in subgroups stratified by them. However, our data revealed that unmarried patients, especially for widowed ones, had a significant survival disadvantage persisting at each sex and age subgroup and all stages of cancer compared with the married subjects. The phenomena suggested that the benefits of marriage on all outcomes evaluated in subgroup analyses had additional underlying etiologies, except for the hypotheses related to the above mentioned risk factors. A widely accepted explanation of why married people had lower mortality from cancer is with regard to a better socioeconomic status, which has been postulated to buffer the effects of stressful events 27, 28. Chronic stress may elicit prolonged secretion of cortisol 29, which triggers a counter‐regulatory response of white blood cells by downregulating their cortisol receptors. This downregulation, in turn, reduces the cell capacity to respond to anti‐inflammatory signals and allows cytokine‐mediated inflammatory processes to flourish 30, which have been validated as poor prognostic factors in cancers 31, 32. Married persons may also enjoy greater financial resources than those who are widowed or divorced due to gains from economies of scale. Evidence for this correlation between marriage and socioeconomic status is widespread throughout the world, and better socioeconomic status has the potential to allow for decreased non‐medically related stress, due to increasing access to healthcare, better food, and better housing 33. In contrast, the diagnosis of cancer in widowed persons, who have no partner to share emotional burden and provide appropriate social support, may result in more distress, depression, and anxiety than their married counterparts. Of note, none of previous studies were designed to address the mechanisms whereby poor socioeconomic status contributed to increased mortality; thus, the concept is not universally accepted until further investigations on this subject have been warranted.

Although many studies have revealed survival differences between categories of married and unmarried patients, they may be not true across the board. In present study, multivariate analyses showed that the survival advantage of marriage was lost to never married individuals; additionally, when non‐married patients were further classified into never married, divorced, and widowed groups, never married or divorced group did not carry the worse survival outcomes for cancer compared to the widowed in subgroup analyses. Being divorced predicted significantly higher risk for mortality in each sex, elderly patients, and stage IV cancers, while the difference of survival between the never married and married group was even not apparent in any stratification. This suggested that marriage itself may increase the likelihood of early diagnosis and treatment of cancer at a young age, or correlate with decreased rates of depression, distress, and anxiety, but it does not independently affect survival in DTC patients.

Albeit this study is both enlightening and relevant to clinical practice; it had several limitations. First, the SEER database only provides the marital status at diagnosis. A few studies have found that the excess all‐cause mortality of the unmarried has increased over time, and the same pattern has been shown for some specific causes of death 34. In the same way, whether the marital status changed after diagnosis is unknown, and this change may also affect the survival. Second, the lack of detailed patient information, such as patient financial status, the existence of preexisting conditions, and mental health, prevents a subset of potential confounders from being accounted for in our analysis as well as disallows us to propose specific mechanisms on why marriage offers increased survival in SEER database. Finally, considering the favorable prognosis of DTC patients, the absence of information on recurrence or distant metastasis after initial therapy in the SEER database limits the evaluation of the effect of marital status on recurrence‐free survival. While this study remains the first of its kind analyzing the effects of marital status on survival in DTC in the American population, each of these limitations must be addressed in future studies to fully decipher what effect marriage truly has on DTC prognosis.

In summary, the data are consistent with the hypotheses mentioned above that marriage somehow protects the patients. An alternative explanation is that although marriage by itself is not protective (i.e., no decreased risk for CSS than never married), once you get married, getting a divorced or become widowed becomes hardship, both physical and psychological. This may be a better explanation. From a societal‐public health standpoint, the first hypothesis has implication of encouraging marriage as a public health measure, whereas, the second hypothesis would discourage divorce and encourage remarriage after widowhood.

Despite these potential limitations, our study had separated non‐married patients based on being never married, divorced, and widowed, and we analyzed each sex, age, and cancer stage individually with a series of traditional risk factors taken into account. Our results confirmed that the unmarried status has an independent impact on cancer‐specific mortality with a varied risk compared with married status. Moreover, we indicated that the unmarried patients groups were heterogeneous, and the widowed patients were always at the highest risk of death of cancer compared with those in other groups. Elderly age, advanced cancer stage, and psychosocial factors may be the reasons for poor survival outcomes in widowed or divorced patients. Physicians caring for these should be aware of their poorer outcomes. Social support systems should provide adequate care and interventions for these patients to help reduce the significant survival differences between married and unmarried patients with DTC, especially for widowed patients.

## Ethics Statement

This study was in compliance with the Helsinki Declaration. An independent ethics committee/institutional review board at Fudan University Shanghai Cancer Center approved our study. Data released from the SEER database does not require informed patient consent because it contains no identifiers and is publicly available. We obtained permission to access the research data file in the SEER program by National Cancer Institute, USA and the reference number was 13579‐Nov2014.

## Conflict of Interest

The authors declare that there is no conflict of interest that could be perceived as prejudicing the impartiality of the research reported.

## References

[1] Siegel, R. , J. Ma , Z. Zou , and A. Jemal . 2014 Cancer statistics, 2014. CA Cancer J. Clin. 64:9–29. 24399786 10.3322/caac.21208

[2] Harness, J. K. , M. K. McLeod , N. W. Thompson , W. C. Noble , and R. E. Burney . 1988 Deaths due to differentiated thyroid cancer: a 46‐year perspective. World J. Surg. 12:623–629. 3245216 10.1007/BF01655866

[3] Mazzaferri, E. L. , and S. M. Jhiang . 1995 Differentiated thyroid cancer long‐term impact of initial therapy. Trans. Am. Clin. Climatol. Assoc..106: 151–168; discussion 168‐170. 7483170 PMC2376543

[4] Davies, L. , and H. G. Welch . 2006 Increasing incidence of thyroid cancer in the United States, 1973‐2002. JAMA 295:2164–2167. 16684987 10.1001/jama.295.18.2164

[5] Torre, L. A. , F. Bray , R. L. Siegel , J. Ferlay , J. Lortet‐Tieulent , and A. Jemal . 2015 Global cancer statistics, 2012. CA Cancer J. Clin. 65:87–108. 25651787 10.3322/caac.21262

[6] Hayat, M. J. , N. Howlader , M. E. Reichman , and B. K. Edwards . 2007 Cancer statistics, trends, and multiple primary cancer analyses from the surveillance, epidemiology, and end results (SEER) program. Oncologist 12:20–37. 17227898 10.1634/theoncologist.12-1-20

[7] Siegel, R. L. , K. D. Miller , and A. Jemal . 2015 Cancer statistics, 2015. CA Cancer J. Clin. 65:5–29. 25559415 10.3322/caac.21254

[8] Tarasova, V. D. , and R. M. Tuttle . 2016 A Risk‐adapted approach to follow‐up in differentiated thyroid cancer. Rambam Maimonides Med. J.. 7:1–10 10.5041/RMMJ.10231 PMC4737510 26886955

[9] Omry‐Orbach, G . 2016 Risk stratification in differentiated thyroid cancer: an ongoing process. Rambam Maimonides Med. J. 7:1–9. 10.5041/RMMJ.10230 PMC4737509 26886959

[10] Antonelli, A. , C. Ferri , S. M. Ferrari , A. Di Domenicantonio , D. Giuggioli , D. Galleri , et al. 2016 Increased risk of papillary thyroid cancer in systemic sclerosis associated with autoimmune thyroiditis. Rheumatology (Oxford) 55:480–484. 26424836 10.1093/rheumatology/kev358

[11] Stansifer, K. J. , J. F. Guynan , B. M. Wachal , and R. B. Smith . 2015 Modifiable risk factors and thyroid cancer. Otolaryngol. Head Neck Surg. 152:432–437. 25552593 10.1177/0194599814564537

[12] Inverso, G. , B. A. Mahal , A. A. Aizer , R. B. Donoff , N. G. Chau , and R. I. Haddad . 2015 Marital status and head and neck cancer outcomes. Cancer 121:1273–1278. 25524565 10.1002/cncr.29171

[13] Aizer, A. A. , M. H. Chen , E. P. McCarthy , M. L. Mendu , S. Koo , T. J. Wilhite , et al. 2013 Marital status and survival in patients with cancer. J. Clin. Oncol. 31:3869–3876. 24062405 10.1200/JCO.2013.49.6489 PMC4878087

[14] Warren, J. L. , C. N. Klabunde , D. Schrag , P. B. Bach , and G. F. Riley . 2002 Overview of the SEER‐medicare data: content, research applications, and generalizability to the United States elderly population. Med. Care 40:IV–3‐18. 10.1097/01.MLR.0000020942.47004.03 12187163

[15] Cooper, D. S. , G. M. Doherty , B. R. Haugen , R. T. Kloos , S. L. Lee , S. J. Mandel , et al. 2009 Revised American thyroid association management guidelines for patients with thyroid nodules and differentiated thyroid cancer. Thyroid 19:1167–1214. 19860577 10.1089/thy.2009.0110

[16] Sobin, L. H. G. M. , and C. H. Wittekind . 2009 UICC: TNM classification of malignant tumors, 7th ed. Wiley‐Liss, New York.

[17] Edge, S. B. , and C. C. Compton . 2010 The American joint committee on cancer: the 7th edition of the AJCC cancer staging manual and the future of TNM. Ann. Surg. Oncol. 17:1471–1474. 20180029 10.1245/s10434-010-0985-4

[18] Gill, R. D. 1992 Multistate life‐tables and regression models. Math Popul. Stud. 3:259–276. 12343718 10.1080/08898489209525345

[19] Cady, B. , C. E. Sedgwick , W. A. Meissner , M. S. Wool , F. A. Salzman , and J. Werber . 1979 Risk factor analysis in differentiated thyroid cancer. Cancer 43:810–820. 427722 10.1002/1097-0142(197903)43:3<810::aid-cncr2820430306>3.0.co;2-b

[20] Qu, N. , L. Zhang , Q. H. Ji , Y. X. Zhu , Z. Y. Wang , Q. Shen , et al. 2014 Number of tumor foci predicts prognosis in papillary thyroid cancer. BMC Cancer 14:914. 25471041 10.1186/1471-2407-14-914 PMC4289292

[21] Goodwin, J. S. , W. C. Hunt , C. R. Key , and J. M. Samet . 1987 The effect of marital status on stage, treatment, and survival of cancer patients. JAMA 258:3125–3130. 3669259

[22] Ghazali, S. M. , Z. Othman , K. C. Cheong , L. K. Hock , W. R. Wan Mahiyuddin , M. A. Kamaluddin , et al. 2013 Non‐practice of breast self examination and marital status are associated with delayed presentation with breast cancer. Asian Pac. J. Cancer Prev. 14:1141–1145. 23621202 10.7314/apjcp.2013.14.2.1141

[23] Brusselaers, N. , F. Mattsson , A. Johar , A. Wikman , P. Lagergren , J. Lagergren , et al. 2014 Marital status and survival after oesophageal cancer surgery: a population‐based nationwide cohort study in Sweden. BMJ Open 4:e005418. 10.1136/bmjopen-2014-005418 PMC4054621 24907248

[24] Lagergren, J. , G. Andersson , M. Talback , S. Drefahl , E. Bihagen , J. Härkönen , et al. 2016 Marital status, education, and income in relation to the risk of esophageal and gastric cancer by histological type and site. Cancer 122:207–212. 26447737 10.1002/cncr.29731

[25] Li, Q. , L. Gan , L. Liang , X. Li , and S. Cai . 2015 The influence of marital status on stage at diagnosis and survival of patients with colorectal cancer. Oncotarget 6:7339–7347. 25749515 10.18632/oncotarget.3129 PMC4466689

[26] Tyson, M. D. , P. E. Andrews , D. A. Etzioni , R. G. Ferrigni , M. R. Humphreys , S. K. Swanson , et al. 2013 Marital status and prostate cancer outcomes. Can. J. Urol. 20:6702–6706. 23587510

[27] Du, K. L. , K. Bae , B. Movsas , Y. Yan , C. Bryan , and D. W. Bruner . 2012 Impact of marital status and race on outcomes of patients enrolled in radiation therapy oncology group prostate cancer trials. Support. Care Cancer 20:1317–1325. 21720747 10.1007/s00520-011-1219-4

[28] Giese‐Davis, J. , A. Waller , L. E. Carlson , S. Groff , L. Zhong , E. Neri , et al. 2012 Screening for distress, the 6th vital sign: common problems in cancer outpatients over one year in usual care: associations with marital status, sex, and age. BMC Cancer 12:441. 23031647 10.1186/1471-2407-12-441 PMC3528655

[29] McEwen, B. S. 2007 Physiology and neurobiology of stress and adaptation: central role of the brain. Physiol. Rev. 87:873–904. 17615391 10.1152/physrev.00041.2006

[30] Miller, G. E. , S. Cohen , and A. K. Ritchey . 2002 Chronic psychological stress and the regulation of pro‐inflammatory cytokines: a glucocorticoid‐resistance model. Health Psychol. 21:531–541. 12433005 10.1037//0278-6133.21.6.531

[31] Formica, V. , J. Luccchetti , D. Cunningham , E. C. Smyth , P. Ferroni , A. Nardecchia , et al. 2014 Systemic inflammation, as measured by the neutrophil/lymphocyte ratio, may have differential prognostic impact before and during treatment with fluorouracil, irinotecan and bevacizumab in metastatic colorectal cancer patients. Med. Oncol. 31:166. 25148896 10.1007/s12032-014-0166-6

[32] Hamilton, T. D. , D. Leugner , K. Kopciuk , E. Dixon , F. R. Sutherland , and O. F. Bathe . 2014 Identification of prognostic inflammatory factors in colorectal liver metastases. BMC Cancer 14:542. 25069793 10.1186/1471-2407-14-542 PMC4125702

[33] Harvei, S. , and O. Kravdal . 1997 The importance of marital and socioeconomic status in incidence and survival of prostate cancer. An analysis of complete Norwegian birth cohorts. Prev. Med. 26:623–632. 9327469 10.1006/pmed.1997.0153

[34] Kravdal, H. , and A. Syse . 2011 Changes over time in the effect of marital status on cancer survival. BMC Public Health 11:804. 21999466 10.1186/1471-2458-11-804 PMC3206482

